# Emergency treatment of food anaphylaxis: a report of 152 cases registered by the Allergy Vigilance Network

**DOI:** 10.1186/2045-7022-3-S3-O1

**Published:** 2013-07-25

**Authors:** A Moneret-Vautrin

**Affiliations:** 1 Nancy University, Vandoeuvre les Nancy, France

## Background

International guidelines are in place for the management of severe food anaphylaxis. Their implementation in emergency services is examined to identify the points needing improvement.

## Methods

One hundred and fifty-two cases of severe anaphylaxis were reported to the Allergy Vigilance Network in France and Belgium in 2011. Information was recorded on a standardised form. A thorough analysis was then undertaken.

## Results

78 paediatric and 74 adult cases were reported. Two deaths occurred. Personnel and emergency centres involved in the management were known in 147 cases, and treatment in 136 cases. First-aid was provided at home by patients themselves on 43 occasions or by the family physician 12 times. 49 called for an ambulance. 89 required hospitalisation in an Emergency Department. Epinephrine auto-injectors were used by 4 patients only. Medicalized ambulances treated 23 patients, and used epinephrine in 11/23 cases. Emergency departments treated 58 patients and used epinephrine in 19 cases. When epinephrine had been injected before arriving at the ED, no further injection in hospital was necessary. In total, epinephrine was given to 37 patients (27.2%). The observation period was much shorter than recommended in the Directives. Patients were discharged without care summaries. Allergists implemented school management plans for all children. 68 to 80% of patients were prescribed self-injectable epinephrine.

**Table 1:** Clinical features of severe food anaphylaxis treated by epinephrine.

Clinical features	% paediatric population 78 cases	% adult population 74 cases	% total population 152 cases	% treatment by epinephrine 37 cases
**Anaphylactic shock**	8.9	24.2	16.4	54.2
**Systemic reaction**	66,6	51.3	59.2	16.3
**Acute asthma**	2.6	5.4	3.9	66.7
**Laryngeal angioedema**	11.5	12.2	11.8	16.7

## Conclusion

Personal care plans including patient education on the use of epinephrine should be more widely used. The frequent prescription of self-injectable epinephrine to the patients is almost at no use. As patients fail to use them when necessary, paramedics should be equipped with auto-injectors and trained to identify severe symptoms. We recommend improved coordination between emergency doctors, paediatricians and allergists.

## Disclosure of interest

None declared.
